# MCL1 binding to the reverse BH3 motif of P18INK4C couples cell survival to cell proliferation

**DOI:** 10.1038/s41419-020-2351-1

**Published:** 2020-02-28

**Authors:** Robert H. Whitaker, William J. Placzek

**Affiliations:** Department of Biochemistry and Molecular Genetics, University of Alabama at Birmingham, Birmingham, AL USA

**Keywords:** Proteins, Apoptosis, Checkpoints, Cell signalling

## Abstract

Commitment to cell cycle entry and cellular duplication is a tightly coordinated and regulated process. Once initiated, a series of multiple checkpoints ensure both accurate genomic replication and chromosomal separation. In the event of unsuccessful cell division, parallel pathways exist that induce the cell to undergo programmed cell death, or apoptosis. At the center of such stress-induced, intrinsic apoptotic regulation lies the BCL2 family of pro- and anti-apoptotic regulatory proteins. In a proliferative state the balance of pro- and anti-apoptotic signaling proteins would be expected to favor an excess population of anti-apoptotic members. While the anti-apoptotic BCL2 family member, MCL1, has been identified to oversee mitotic progression, direct communication between the BCL2 family and cell proliferation has not been observed. In this study, we demonstrate a direct protein–protein interaction between MCL1 and the G_1_/S checkpoint protein, P18INK4C. This interaction is mediated by a reverse BH3 (rBH3) motif located in P18INK4C’s C-terminal ankyrin repeat. MCL1 is further shown to decrease P18INK4C expression and thereby regulate cell cycle entry in a retinoblastoma (RB1)-dependent manner. Our findings establish a mechanism for translation independent and direct communication between the BCL2 family regulation of apoptosis and CDK4/6-RB regulation of early G_1_/S transition during cellular division/growth.

## Introduction

Cellular response to intrinsic stress, including DNA damage, metabolic imbalance, and starvation, first results in an attempt to repair or correct the problem and when correction is unsuccessful leads to induction of programmed cell death or apoptosis. Multiple dedicated signaling events and mechanisms have been characterized to govern these cell fate decisions and when unsuccessful, promote transcription factor activation and expression of key pro-apoptotic proteins. These pro-apoptotic proteins ultimately interface with a single protein family, the B-cell lymphoma 2 (BCL2) family of pro- and anti-apoptotic proteins, which serve as the gatekeepers of apoptosis^1^. The BCL2 family is subdivided into three functional groups: (1) pro-apoptotic effectors (BAK, BAX, BOK); (2) anti-apoptotic proteins (BCL2, BCLxL, BCLW, MCL1, BFL1/A1); and (3) BH3-only pro-apoptotic proteins (e.g*.*, BIM, BAD, BID, PUMA, NOXA). The BCL2 family regulates apoptosis through a series of interactions between these subfamilies using a shared BCL2 homology 3 (BH3) amphipathic alpha-helical motif that is common to all BCL2 family members (reviewed in refs. ^2,3^). This BH3 motif lies at the center of BCL2 family regulation with the anti-apoptotic family members positioned to bind to BH3-motifs of the pro-apoptotic subfamilies and sequester them in inactive dimeric complexes. When there is insufficient expression of anti-apoptotic BCL2 family members to sequester the pro-apoptotic effectors, then BAK and BAX homo-oligomerize in mitochondrial outer membranes. This initiates mitochondrial outer membrane permeabilization (MOMP), release of cytochrome C, and caspase activation. Two models have emerged that detail how these interactions ultimately induce oligomerization of the pro-apoptotic effectors through their BH3 motifs, though in each model, oligomerization can be suppressed through anti-apoptotic BCL2 binding to the BH3 helix of BAK or BAX rendering them unable to oligomerize. Separately, the BH3-only family members act either to promote BAK/BAX oligomerization or to sequester anti-apoptotic BCL2 proteins. In all models, function of the BCL2 family is regulated by the interactions between anti-apoptotic proteins and BH3 helices of pro-apoptotic proteins as these interactions serve as a fulcrum between cellular viability and apoptosis initiation^4,5^.

The central positioning of the anti-apoptotic BCL2-family proteins in regulating cell death, specifically cellular response to intrinsic stress, has situated them as key regulators of tumorigenesis and/or anti-cancer therapeutic response. Upregulation of the anti-apoptotic BCL2 family members in cancers is a common event with various cancer types employing one or a subset of these proteins to tolerate the genomic and energetic stress that often accompanies tumorigenesis^6^. Overexpression of one of the anti-apoptotic BCL2 family proteins, MCL1, has been identified as a mechanism utilized by cancers to evade a number of standard chemotherapies, including taxanes, vinca alkaloids, platinum containing compounds, and radiation^5,7–11^. More recently, BH3 mimetics have been developed to suppress anti-apoptotic BCL2 family proteins as a method of treating this key mechanism associated with cancer survival^12–14^. Thus, interactions with the anti-apoptotic BCL2 family are well recognized for their importance in regulating acute response to cellular stress. Owing to MCL1’s positioning in cancer, we initially sought to identify peptides capable of specifically targeting MCL1. These studies led to our identification of a novel sequence motif, a reversal of the traditional BH3 motif, which we termed a reverse BH3 (rBH3) motif as it retains key consensus acidic and hydrophobic residues^15^. This sequence highlighted the importance of targeting the P2 pocket to gain MCL1 specificity and further provided the possibility that other proteins may directly impact BCL2 family regulation^15^. One protein that was identified to putatively contain a rBH3 motif is the G_1_/S cell cycle regulator, P18 (P18INK4C, CDKN2C). Here, we demonstrate that the rBH3 motif is more than a unique peptide sequence, but that it is a natural protein motif that is able to mediate direct protein-protein interactions between MCL1 and a rBH3-containing protein.

## Materials and methods

### Protein expression

Human MCL1 [Uniprot: Q07820] (residues 163–326), human P18 [Uniprot: P42773] (residues 1–168), A1–4 (P18 residues 1–140), A4–5 (P18 residues 106–168), P16 [Uniprot: P42771] (residues 1–156), and AC (P16 residues 1–113 and P18 residues 106–168) were sequence-optimized for bacterial expression and cloned into the restriction sites of NdeI and HindIII in a pET28a vector (EMD Millipore) to incorporate a N-terminal hexa-histidine (His6) tag and transformed into BL21(DE3) E. Coli (New England Bio Labs). Cultures were grown under kanamycin selection in Luria broth to a 600 nm optical density of 0.6 and expression was induced by addition of 1 mM isopropyl β-d-1-thiogalactopyranoside (Thermo Fisher). Cultures were harvested 3–4 h after induction by centrifugation at 4700 × *g*. Resulting bacterial pellets were stored at −80 °C. His6-MCL1 bacterial pellets were re-suspended (10 mL/wet g of pellet) in Buffer A (1 × PBS, pH 6.8) with the addition of 1× lysozyme (0.25 mg/mL) (Thermo Fisher) and 1 protease inhibitor tablet (Pierce #88266), sonicated, and cleared at 14,000 × *g*. Resulting lysate was purified on NGC FPLC (Bio-Rad Laboratories) using a 1 mL HiTrap HP nickel column (GE Healthcare) equilibrated with Buffer A. Protein was eluted with a 2 mM–1000 mM imidazole gradient. His-MCL1 containing fractions, as identified by denaturing polyacrylamide gel electrophoresis, were further purified by gel filtration in Buffer B (1× PBS, pH 6.8) on a HiPrep 16/60 Sephacryl S-100 column (GE Healthcare). Resulting protein identity was confirmed by matrix-assisted laser desorption/ionization (MALDI) mass spectrometry.

### Peptide synthesis

Peptides were synthesized using a standard, double-addition, fluorenylmethyloxycarbonyl (FMOC), solid-phase peptide synthesis strategy on the Prelude system (Gyros Protein Technologies). 4-(2′,4′- dimethoxyphenyl-fmocaminmethyl)-phenoxyacetamido-methylbenzhydryl amine resin (rink amide MBHA resin, Anaspec) was swelled in N,N-dimethylformamide (DMF, Fisher Scientific) followed by methylene chloride (DCM, Fisher Scientific) to increase surface area availability for bonding. Using a double-addition FMOC strategy, the N-terminal FMOC on the growing peptide chain was deprotected with 0.8 M piperidine (Fisher Scientific) in DMF for 2 min and 30 s, then the following amino acid (200 mM) to be added to the N-terminus was activated with 0.4 M O-(1H-6-Chlorobenzotriazole-1-yl)-1,1,3,3-tetramethyluronium hexafluorophosphate (HCTU, Anaspec) in DMF for nucleophilic attack of the N-terminal peptidyl-resin. Next, 800 mM 4-Methylmorpholine (NMM, Fisher Scientific) in DMF was added, and the peptidyl-resin, HCTU, NMM slurry was mixed for 30 min followed by 4 × 30 s DMF washes. Peptidyl-resin was cleaved using 88% trifluoroacetic acid, 5% Water, 5% phenol, and 2% triisopropylsilane for 180 min. The cleaved peptide was filtered by hand using the Prelude reaction vessels away from the resin. Filtered, cleaved peptide was cold-ether precipitated and centrifuged at 14,000 × *g* to pellet the resin-cleaved, crude peptide. Crude peptides were lyophilized and re-suspended in 80% water/20% acetonitrile and purified over a Zorbax Eclipse XDB-C18 column (Agilent) on a 1260 Infinity HPLC (Agilent) with a 5–60% acetonitrile gradient. Peptide mass was confirmed by MALDI-MS. Peptide concentration was determined by one-dimensional (1D) nuclear magnetic resonance (NMR) comparing approximated peptide concentrations to known standards. The N-terminus of BAK peptidyl-resin was conjugated with a 6-aminocaproic acid (AHX) linker by two 30-min double additions as described above. Finally, the FITC fluorophore was coupled to the AHX-peptide-resin in the dark under nitrogen with two 30 min additions in a thick slurry of 8 mg FITC in Pyridine/DMF/DCM (12:7:5) followed by subsequent cleavage, purification, and verification.

### Fluorescence polarization anisotropy (FPA)

A FPA assay was developed and characterized by experiments performed in black, non-treated 96-well microplates (Nunc #12–566–23). Experiments were performed using technical triplicates with three biologic replicates collected on separate days and with separate protein preparations. Reactions were conducted in 1x PBS at pH 7.4 in 100 µL reaction volumes. Initially, 100 nM His6-MCL1 protein and variable unlabeled P18x peptides or dimethyl sulfoxide (DMSO) were added to the well and incubated at room temperature (RT) for 10 min. Then 10 µL of 100 nM FITC-AHX-BAK (final concentration 10 nM) was added and samples incubated for an additional 1 h, shaking at RT in the dark. Plates were read on a Victor X5 (Perkin Elmer) plate reader using the FP-Fluorescein(1.0 s) setting (CW-lamp energy, 65535; CW-lamp Filter, F485-slot A5; Emission Filter, F535-Slot A5; 1 s counting time; and G factor, 1. Curve fitting was performed using prism software (Graphpad software, Inc) using the equation, *Y* = Bottom + (Top-bottom)/(1 + 10^((LogIC50-X)*HillSlope)).

### Cell culture

PC-3, WAC2, HELA, MDA-MB-231, and DU-145 cells were maintained in RPMI-1640 medium supplemented with 10% fetal bovine serum, 2.05 mM l-glutamine, 100 units per mL each of penicillin and streptomycin, and 0.25 μg/mL of Fungizone antimycotic (Life Technologies) in a humidified atmosphere with 5% CO2. Validation of cell lines was completed using short tandem repeat profiling against published ATCC signatures. Silencer Select validated small-interfering RNAs (siRNAs) and negative control siRNAs were purchased from Ambion. The siRNA product IDs are: si-MCL1 (s8583), P18(118622), and negative control, siGFP (AM4626) was used with no significant sequence similarity to human gene sequences. cDNA for P18 (clone ID: 3907917) and MCL1 (clone ID: 3138465) was obtained from Life Technologies and the coding sequence was cloned into the pcDNA3.1 plasmid for exogenous expression. siRNA and plasmid transfections were carried out using Lipofectamine RNA iMAX reagents and Lipofectamine 3000 reagents (Life Technologies), respectively. Cells were harvested for analysis after 48 or 72 h transfection as designated in text. For CellTrace™ Violet staining experiments cells (1 × 106/mL) were stained with 100 nm CTV, C34557, purchased from Thermo Fisher Scientific then plated, and transfected after 24 h.

### Chemicals

ABT-199 (S8048), leupeptin hemisulfate (57830), and calcitriol (S1466) were purchased from Selleck Chemicals. S63845 was purchased from Chemietek.

### Quantitative reverse transcription PCR (RT-qPCR)

Total RNA was isolated from cells by Trizol reagent (Life Technologies) and purified by PureLink RNA Mini Kit (Ambion). Genomic DNA was removed using RNAse-free DNAse treatment. Final RNA concentrations were measured by absorbance at 260 nm and quality was confirmed using a A260/280 ratio of ~2.0. cDNA was prepared using 1 μg of total RNA in 20 μL reverse transcription reaction with qScript cDNA SuperMix (Quanta Biosciences) according to manufacturer’s protocol. RT-qPCR reactions were performed in a 10 μL reaction containing 4 μL of the diluted complementary DNA (cDNA), 5 μL PerfeCta SYBR green FastMix, Low ROX (Quanta Biosciences), and 0.5 μL each of forward and reverse primers at final concentrations of 250 nM. All qPCR reactions were run in quadruplicate on MicroAmp Optical 384-well plates on a ViiA 7 instrument (Applied Biosystems). Amplification conditions consisted of the initial denaturation step at 95 °C for 3 min, followed by 40 cycles of 10 s at 95 °C, and 30 s at 60 °C. Afterwards, melting curves were generated to confirm presence of a single uniform peak. Results were analyzed in ViiA 7 software by comparative CT (ΔΔCT) method using GAPDH as a normalization control and exported for analysis and presentation in GraphPad Prism (GraphPad Software, La Jolla, CA, USA). The following primers (5′–3′) were used:

*MCL1*: (F) GGACATCAAAAACGAAGACG and (R) GCAGCTTTCTTGGTTTATGG; GAPDH: (F) CCACATCGCTCAGACACCAT and (R) CCAGGCGCCCAATACG; P18: (F) GGGGACCTAGAGCAACTTA and (R) CAGCGCAGTCCTTCCAAAT.

### Protein extraction and western blot

Whole-cell lysates were prepared by lysing cells on ice with 1× RIPA buffer (Boston BioProducts): 50 mM Tris-HCl pH 7.4, 150 mM NaCl, 1% NP40, 0.5% sodium deoxycholate and water (rest) supplemented with protease inhibitors (1 mM AEBSF, 0.8 μM aprotinin, 0.05 mM bestatin, 0.015 mM E-64, 0.02 mM leupeptin, 0.01 mM pepstatin A). Protein lysates were resolved by sodium dodecyl sulfate–polyacrylamide gel electrophoresis and transferred to polyvinylidene fluoride membrane in a wet transfer system for 1 h at 100 V. Membranes were incubated with anti-MCL1 Abs (D2W9E and D35A5, Cell Signaling), anti-BAX Abs (D2E11, Cell Signaling), anti-P18 Abs, DCS-118 from Cell Signaling, anti-β-actin Abs, PA1–21167, from Pierce (Rockford, IL, USA), and anti-CDK6 Ab, (C-21): sc-177 from Santa Cruz at a dilution of 1:1000, at 4 °C overnight. HRP-conjugated secondary Abs were used for detection using ECL2 reagent (Pierce). Immunoblots were visualized on Bio-Rad ChemiDoc MP imaging system.

### Flow cytometry

PC-3, WAC2, MDA-MB-231, or DU-145 cells (2 × 10^5^) were transfected with control siRNA or si-P18 alone or si-MCL1 alone. PC-3, WAC2, MDA-MB-231, or DU-145 cells (2 × 10^5^) were transfected with Vehicle or MCL1 plasmid for alone for 48 h or for 30 h then treated with S63845 or ABT-199, final DMSO concentration 0.5% for 18 h. After 18 h for siRNA experiments or 48 h for overexpression experiments, cells were harvested. Cells where harvested for FACS by washing in 1× dPBS (Corning) and treating with trypsin (Gibco) for 5 min at 37 °C. Typsin was inactivated by the addition of RPMI media with FBS and collected cells were centrifuged at 500 rpm for 5 min, media and trypsin aspirated, and washed in 1x PBS and centrifuged at 500 rpm for 5 min, aspirated and the cellular pellet re-suspended in 1× PBS with 0.1% formaldehyde (Fisher) by pipetting. Cells were fixed by adding ice cold 70% ethanol (Fisher) and incubating at −20 °C for 2 h for PI staining cells were pelleted at 1500 rpm for 10 min and washed 2× with PBS, 1 mg/mL RNase A (Thermo Fisher) was added to the cells and incubated at 37 °C for 30 min. In all, 1 × 10^5^ cells were stained with 0.1 mg/mL Propidium Iodide (BD Biosciences, San Jose, CA, USA) at room temperature for 15 min. Then cells were subjected to flow cytometry on BD LSRFortessa FACS, 50,000 events were collected for each sample and data was analyzed by FlowJo V10. For Annexin V/PI experiments, cells were harvested in a similar manner, washed 2x in PBS, re-suspended in 1x Annexin V binding buffer (CBD Pharmingen), and stained with FITC Annexin V (CBD Pharmingen) and PI. Then cells were subjected to flow cytometry on BD LSRFortessa FACS, 50,000 events were collected for each sample and data was analyzed by FlowJo V10. For CellTrace™ Violet stained cells, after 24, 48, or 72 h transfection cells were harvested in a similar manner as PI stained cells, but fixed in ice cold 70% ethanol (Fisher) and incubating at −20 °C for 20 min. Then cells were subjected to flow cytometry on BD LSRFortessa FACS, 50,000 events were collected for each sample and data was analyzed by FlowJo V10. All data were collected with three biologic replicates analyzed on separate days with technical duplicates for each analysis.

### Statistical analyses

All experiments were repeated at least three biologic replicates using two or three technical replicates, as reported, with data expressed as the mean ± S.D. No samples were excluded. Differences between two data sets were calculated using a two-tailed unpaired Student *t*-test with *P* < 0.05 considered statistically significant. Statistical analysis was performed in Prism (Graphpad Inc.) or Microsoft Excel. *P < 0.05, **P < 0.01, ***P < 0.001.

## Results

### P18 binds to MCL1 in vitro

**Fig. 1:**
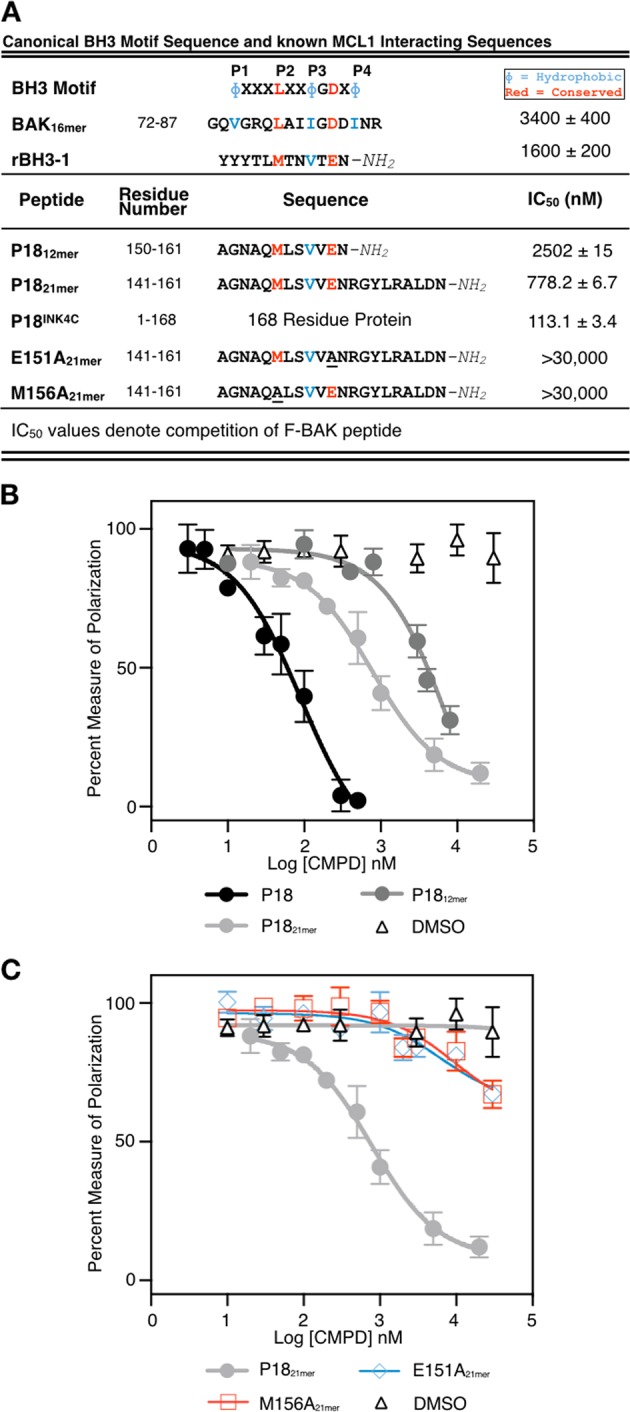
P18 rBH3 sequence inhibits BAK association with MCL1. **a** Table of peptide sequences and IC_50_ values of FPA in 1B. **b** Competition fluorescence polarization anisotropy assay (FPA) of P18 protein and peptides outcompeting F-BAK_23mer_ (10 nM) for MCL1 (100 nM) binding. **c** FPA of P18_21mer_ and mutant peptides outcompeting F-BAK_23mer_ (10 nM) for MCL1 (100 nM) binding. All data *N* = 3.

To test if the rBH3 motif functions as a binding motif with MCL1 in native human proteins, we followed up on BLAST analysis performed on the initial rBH3–1 sequence^15^. This analysis identified a putative rBH3 in the c-terminal alpha-helix of the cell cycle regulatory protein P18 (P18INK4C, CDKN2C). As was observed in rBH3–1 (Fig. 1a), the P18 rBH3 (residues 150–161) contains a homologous substitution wherein the conserved aspartic acid residue in the consensus BH3 sequence is replaced with a glutamic acid residue (E151). Further, neighboring leucine (L155) and methionine (M156) residues are located in the hydrophobic position that makes contact with the P2 pocket of MCL1 while a valine residue is positioned in the P3 hydrophobic position. To confirm that the rBH3 sequence in P18 is able to bind to MCL1 we utilized a competitive fluorescent polarization (FP) assay to assess the ability of P18-derived rBH3 peptides or full-length P18 protein to inhibit binding of a fluorescein-labeled native binding partner, in this case the 23 amino acid BAK BH3 helix (F-BAK). We observed that P18 protein is able to inhibit F-BAK association with MCL1 with an IC_50_ of 113.1 ± 3.4 nM. To localize this binding to the rBH3 motif, we prepared two peptides that are composed by the c-terminal 21 or 12 residues of P18, residues 141–161 (21 mer) or 160–161 (12 mer), respectively. We observed that both the 21-mer and 12-mer peptides, though in non-globular conformations, retained competitive inhibition of F-BAK with IC_50_ values of 778.2 ± 6.7 nM and 2502 ± 15 nM, respectively (Fig. 1b). This decrease in affinity is consistent with prior studies that have looked at the impact that stabilization of BH3-containing alpha helices have on association with anti-apoptotic BCL2 proteins^16^. In this case, the rBH3 sequence in the full-length P18 protein is natively stabilized in the globular fold while the synthesized peptides are not. We then sought to determine the importance of the key acidic and hydrophobic residues that mimic the BH3 sequence by synthesizing alanine substitutions of E151 and M156, respectively. We observed that both mutants induced a significant reduction in binding with IC_50_ values of >30,000 nM (Fig. 1c). These studies demonstrate in vitro association of P18, through its rBH3 sequence, with MCL1 and that such association inhibits F-BAK association with MCL1 in a biologically relevant fashion.

### P18 endogenously associates with MCL1

**Fig. 2:**
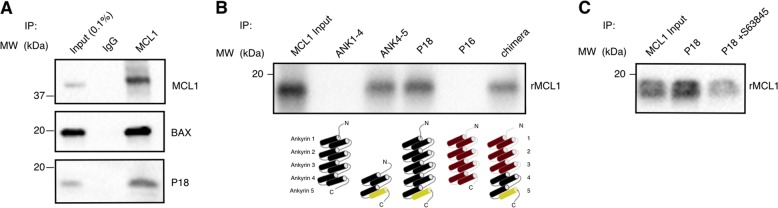
MCL1 binds specifically to P18 through C-terminal rBH3. **a** Western blot of endogenous coIP of MCL1 (IP) with P18 (IB), BAX (IB), and P18 (IB) in WAC2. **b** Western blot of in vitro pulldown of recombinant proteins (IP), ANK1–4, ANK4–5, P18, p16, and chimera, with recombinant MCL1 (rMCL1) (IB). Cartoon of the p16, P18, and chimera proteins are shown below the blot. rBH3-containing helix is highlighted in yellow (Residues: 150–161). **c** Western blot of in vitro pulldown of recombinant P18 (IP) with rMCL1 (IB) with and without MCL1 inhibitor S63845.

After confirming the ability of P18 to bind to MCL1, we next sought to probe the endogenous interaction of P18 with MCL1. We tested for P18 expression in a selection of solid tumor cell lines and chose two, PC-3 (prostate cancer) and WAC2 (neuroblastoma), to further study the interaction based on P18 expression levels. In both cell lines, we probed for and observed the co-immunoprecipitation (coIP) of both P18 and the known MCL1 binding protein BAX following immunoprecipitation (IP) of endogenous MCL1 or control IgG (Fig. 2a and Supplementay Fig. S1A). In PC-3 cells, we were also able to perform the reverse IP of endogenous P18 using a monoclonal antibody specific for P18 or control IgG and probed for and observed coIP of both MCL1 and the known P18 binding protein CDK6 (Supplementary Fig. S1B). These data demonstrate the endogenous interaction between MCL1 and P18 in multiple human-derived cell lines.

### The P18 rBH3 motif is necessary and sufficient to mediate binding

P18 is a member of the INK4 protein family [P16INK4A (P16, CDKN2A), P15INK4B (P15, CDKN2B), P18INK4C (P18, CDKN2C), and P19INK4D (P19, CDKN2D)] that regulate G_1_/S cell cycle progression with all INK4 proteins retaining homologous CDK4/6 inhibitory function. INK4 family proteins have homologous structures containing either four or five ankyrin (ANK) repeats, a helix-turn-helix structural unit known to mediate protein-protein interactions^15,17^. Notably, P15 and P16 are composed of four ankyrin repeats while P18 and P19 have five ankyrin repeat units. The rBH3 in P18 resides in its fifth ankyrin repeat (residues 141–168). To determine if the P18 rBH3 motif is sufficient to mediate binding with MCL1 and that the two do not simply interact through a shared cellular complex, we tested the ability of recombinant and chimeric P18 and P16 proteins to exogenously pull-down recombinant MCL1. Based on prior stability studies of ankyrin repeats within INK4 family proteins^18,19^, we designed two truncations of P18 (Supplementary Table S1). The first, P18-ANK1–4 (ANK1–4), removes the rBH3-containing fifth ankyrin repeat. The second, P18-ANK4–5 (ANK4–5), retains the rBH3 domain as well as ankyrin repeat 4 to facilitate folding. As a negative control, we expressed recombinant P16 that natively contains four ankyrin repeats and does not contain a putative rBH3 motif^17,20^. We observed that only the ANK4–5 and P18 protein constructs (Fig. 2b, lanes 3 and 4), both containing the rBH3 motif found in ANK5, successfully pulled down MCL1 while both the truncation ANK1–4 and P16 proteins (Fig. 2b, lanes 2 and 5) showed no interaction (Fig. 2b). To further demonstrate that the rBH3-containing ANK5 mediates binding, we designed a five ankyrin repeat chimeric protein consisting of P16-ANK1–3 and P18-ANK4–5 (chimera) (Supplementary Table S1). This chimeric protein gained the ability to pulldown recombinant MCL1 (Fig. 2b, lane 6). Finally, to demonstrate that the interaction between P18 and MCL1 utilizes the BH3-binding pocket to mediate the interaction, we employed a recently developed MCL1-specific small-molecule BH3 mimetic, S63845^21^, and tested its ability to disrupt pulldown. Consistent with the rBH3 sequence in P18 binding to the BH3 pocket of MCL1, addition of S63845 to the mixture suppressed the ability of P18 to pull-down MCL1 (Fig. 2c). These data demonstrate that the rBH3-containing ANK5 portion of P18 is both necessary and sufficient to mediate direct association of P18 with MCL1 and inhibition of the BH3 pocket in MCL1 blocks this interaction.

### MCL1 induces loss of P18

When active, CDK4 and CDK6 proteins phosphorylate the tumor suppressor, RB1, which then releases the key S phase entry transcription factor, E2F1^22,23^. Human cancers commonly delete P16 and/or P15 to overcome growth inhibition, yet the rBH3-containing member, P18, whose expression has been shown to compensate for loss of P16, is rarely deleted^24^ (Supplementary Fig. S2). To confirm the ability of the rBH3 motif to mediate direct protein-protein interaction between MCL1 and P18 and elucidate the impact that this binding has on cell growth and viability we chose the neuroblastoma cell line, WAC2, which contains a homozygous deletion of P16, as the main cell model for these studies^25^. We confirmed our results in PC-3 cells, a castration resistant prostate cancer model often used in BCL2 family studies.

**Fig. 3:**
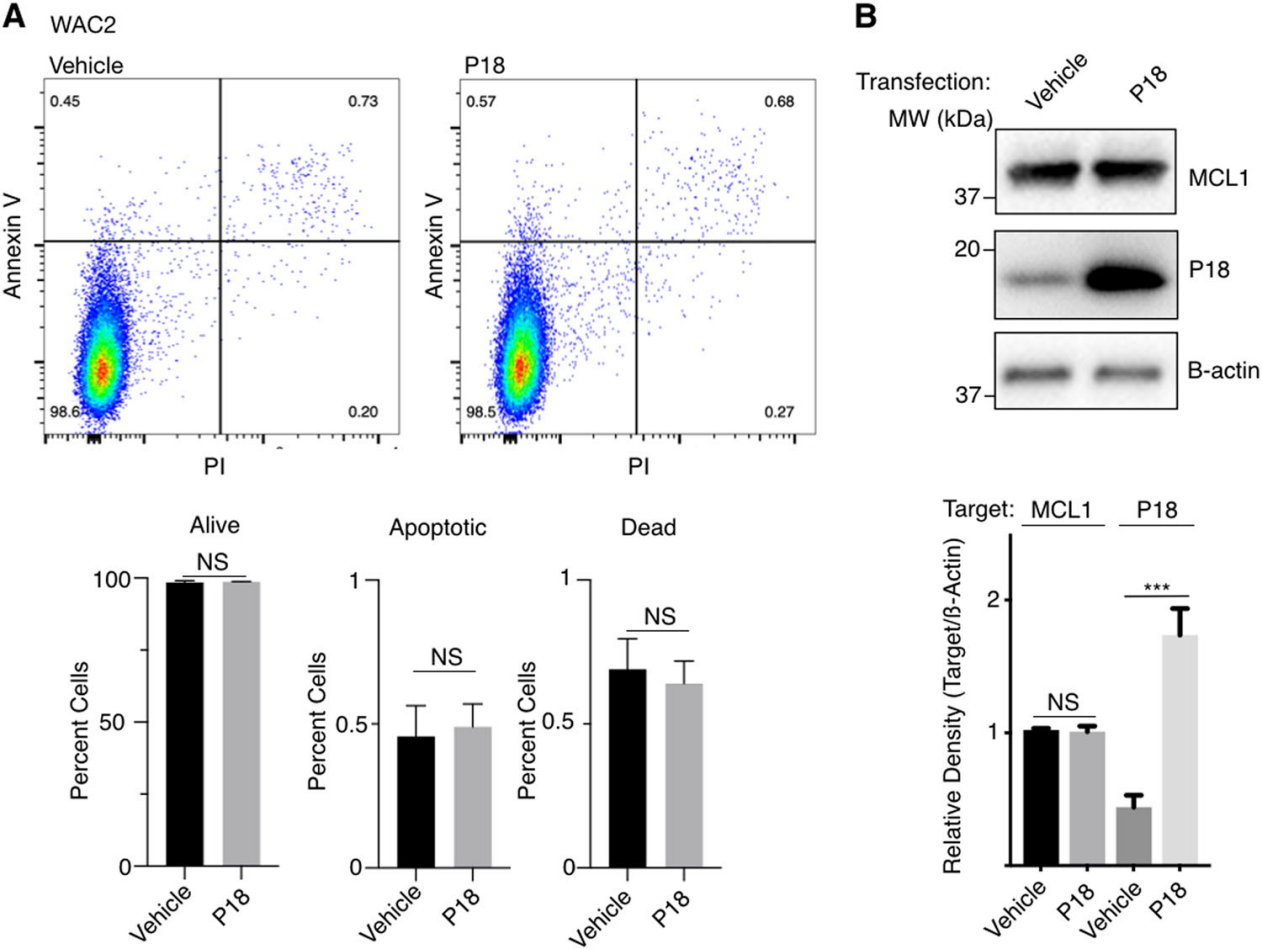
P18 transfection does not induce apoptosis. **a** Transfection of vehicle control (left) or P18 (right) (48 h) in WAC2 with Annexin V and PI staining FACs analysis. **b** Western blot confirming P18 expression transfection and overexpression. All data are presented as mean ± S.D., *N* = 3. The statistical significance was determined by unpaired Student *t*-test where *P > 0.05; **P > 0.01; ***P > 0.001.

Typically, interference of BH3 binding to anti-apoptotic BCL2 family members has been implicated in pro-death signaling and this model serves as the basis for recent development of anti-cancer compounds targeting the BCL2 family^26^. As such, we first sought to determine if increased P18 would induce an apoptotic response. In each of the tested cell lines, we observed that overexpression of P18 showed no evidence of cellular stress or apoptosis induction (Fig. 3 and Supplementary Fig. S3).

**Fig. 4:**
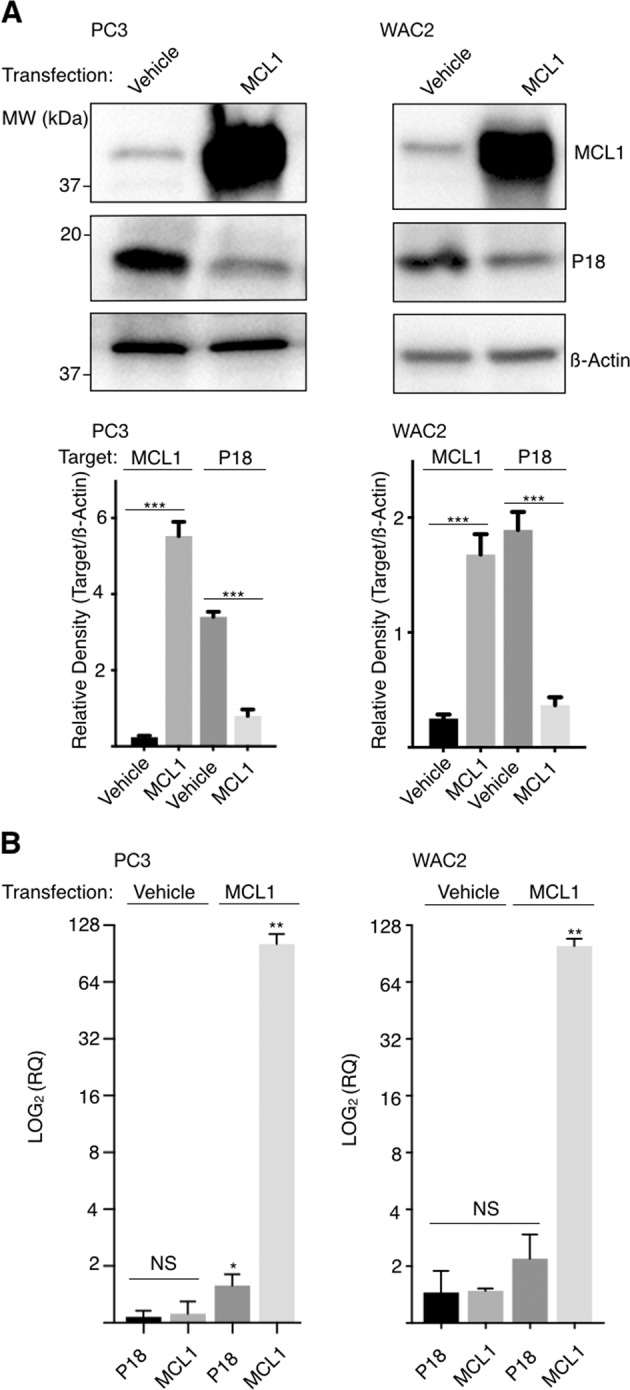
MCL1 negatively affects P18 protein. **a** Western blot of MCL1 transient transfection. MCL1, P18, and actin IB in PC-3 and WAC2 show increase in MCL1 corresponds with decrease in P18 protein. Quantification of band intensity shown below blot. **b** RT-qPCR analysis in PC-3 and WAC2 of MCL1 transient transfection or vehicle control. Expression normalized to GAPDH. All data are presented as mean ± S.D., *N* = 3. The statistical significance was determined by unpaired Student *t*-test where *P > 0.05; **P > 0.01; ***P > 0.001.

**Fig. 5:**
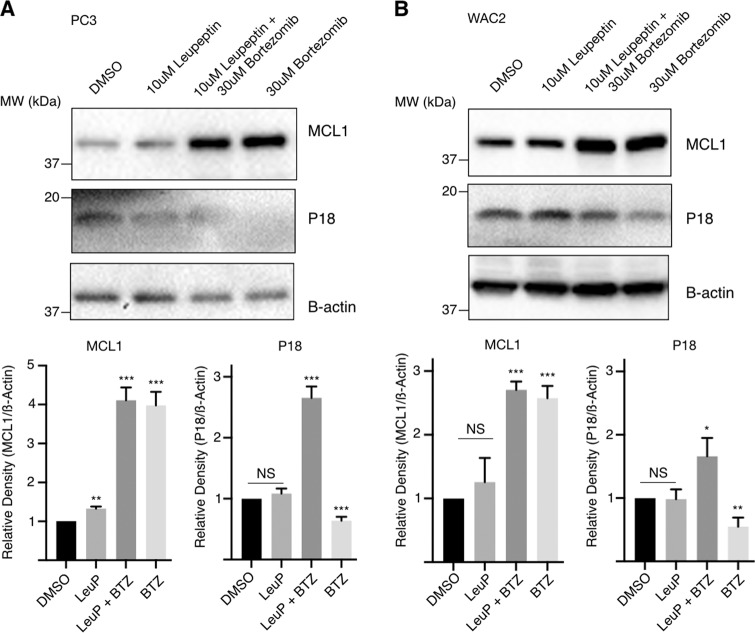
MCL1 impact on P18 degradation. Western blot of DMSO control (0.3%), 10 µM leupeptin (LeuP), 10 µM LeuP + 30µM bortezomib (BTZ), or 30 µM BTZ treated (**a**) PC3 cells or (**b**) WAC2 cells for 4 h. Quantification of band intensity shown below blot. All data are presented as mean ± S.D., *N* = 3. The statistical significance was determined by unpaired Student *t*-test where *P > 0.05; **P > 0.01; ***P > 0.001.

During initial studies to obtain the coIP of P18 and MCL1, we tested a number of MCL1 overexpression conditions prior to immune-precipitation with the MCL1-specific or P18-specific monoclonal antibodies. However, analysis of western blots resulting from these tests led us to suspect that elevated MCL1 protein levels were having a deleterious effect on P18 protein levels. Following this observation, we confirmed that transient transfection and subsequent overexpression of MCL1 results in a decrease of P18 protein (Fig. 4a). We further confirmed that this decrease in P18 protein does not coincide with a decrease of P18 mRNA expression (Fig. 4b). Notably, bortezomib (Velcade, BTZ), a proteasome inhibitor (targeting the chymotrypsin-like and caspase-like enzymatic modalities of the proteasome)^27,28^ does not rescue this effect. In fact, bortezomib treatment caused an increase in MCL1 protein and a subsequent decrease in P18 protein (Fig. 5 and Supplementary Fig. S4). We therefore sought to determine if alternative manipulation of the proteasome could rescue loss of P18. We observed that treatment with the cysteine-protease-specific inhibitor, leupeptin (LeuP), had a marginal to no impact on MCL1 and P18 as a single agent. However, co-treatment of BTZ with LeuP significantly increased the expression of both MCL1 and P18 and rescued the negative effect that single agent BTZ has on P18 (Fig. 5). These data suggest that MCL1 has a transcriptionally independent negative effect on P18 protein that is mediated through a cysteine-protease degradation process.

### MCL1 regulates P18 to promote G_1_/S progression

**Fig. 6:**
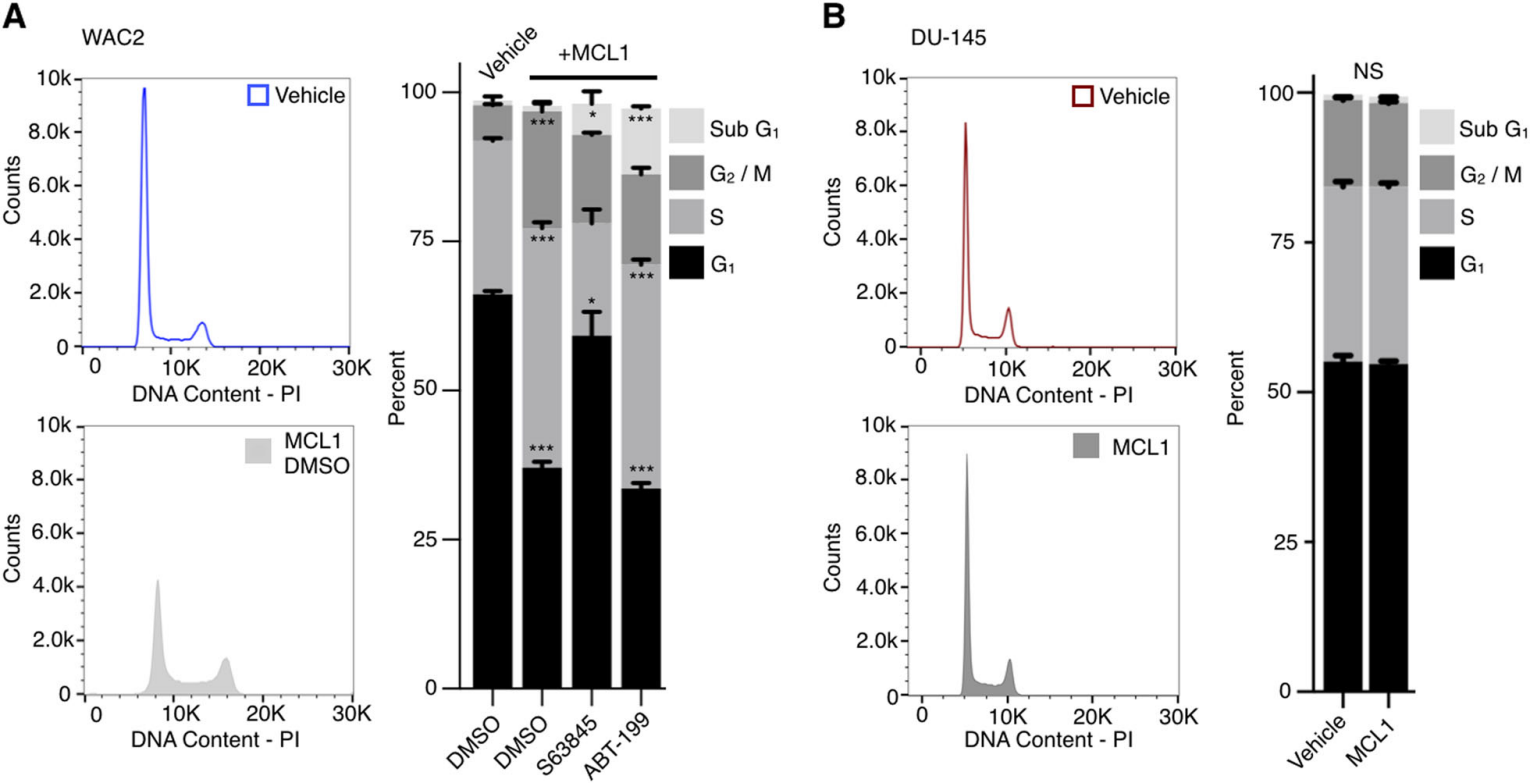
MCL1 modulation of cell cycle is RB dependent. **a** PI chromatograms (left) of vehicle or MCL1 overexpression in the RB1-positive cell line, WAC2. Cell cycle population analysis of chromatographs as well as corresponding studies of MCL1 overexpressed and treated with BH3 mimetics S63845 or ABT-199 (right). **b** PI chromatograms (left), vehicle control and transient MCL1 overexpression in the RB1-mutant cell line, DU-145 with corresponding cell cycle population analysis on right. All data are presented as mean ± S.D., *N* = 3. The statistical significance was determined by unpaired Student *t*-test where *P > 0.05; **P > 0.01; ***P > 0.001.

The established role of P18 as a negative cell cycle regulator in conjunction with our observation that MCL1 mediates depletion of P18 protein, led us to assess how MCL1 overexpression affects the cell cycle. Using propidium iodide (PI) DNA content staining and FACS analysis, we observed that in unsynchronized WAC2 and PC-3 cell lines, overexpression of MCL1 results in a decrease in the G_1_ cell population (Fig. 6a and Supplementary S5) with a corresponding increase in S (PC-3) and G_2_/M (WAC2) populations. To put MCL1 modulation of the cell cycle in perspective of P18, we performed siRNA inhibition to gauge the impact that loss of P18 has on cell cycle populations. We observed that si-P18 exhibited a decrease in G_1_ and increase in cellular proliferation in both WAC2 and PC-3 cell lines (Supplementary Figs. S6 and S7, respectively).

Based on the above studies, we hypothesized that the BH3 pocket of MCL1 was interacting with the rBH3 motif in P18 to induce degradation and thereby promote G_1_/S transition. To confirm that interactions with the BH3-pocket of MCL1 directly modulate the observed G_1_ progression, we employed the MCL1-specific (S63845) and BCL2-specific (ABT-199) small-molecule inhibitors^21,29,30^. Both of these inhibitors act as BH3 mimetics and bind into the BH3 pockets of MCL1 or BCL2, respectively, to suppress interaction with BH3 pocket binding proteins, such as P18 interaction with MCL1. We observed that the MCL1-specific inhibitor rescued the MCL1 overexpression induced decrease in G_1_ population back to control levels (Fig. 6a) while the BCL2-specific inhibitor had no impact on the change in G_1_ population. These data suggest that BH3 interactions between MCL1 and target proteins provide a novel mechanism for free MCL1 to modulate cellular progression through the early INK4 G_1_/S checkpoint. Further, the lack of impact of the BCL2 inhibitor on G_1_/S progression, though with a more significant impact on the sub-G_1_ cell population, demonstrates that this is a MCL1-specific effect and not a general mechanism of BH3-binding proteins.

In order contextualize the effect of MCL1 on G_1_/S cellular transition within the P18 regulated CDK4/6-RB1 pathway, we assessed the impact of that MCL1 overexpression has on DU-145 cells, a P18 expressing but RB1-mutant (non-functional protein through exon 21 deletion) prostate cancer cell line^31^. With the deletion of RB1, we expect that changes in P18 should have no effect on G_1_/S transition. We therefore first confirmed that si-P18 has no effect on the cell cycle population in DU-145 (Supplementry Fig. S6). We then determined that MCL1 overexpression retains the ability to negatively influence P18 protein expression in DU-145 cells (Supplementary Fig. S8). Finally, we assessed the impact of MCL1 overexpression in DU-145 cells and found that MCL1 overexpression induced no changes in the G_1_ population compared to control (Fig. 6b). These data demonstrate that MCL1 regulation of P18 protein levels is not a secondary effect from its impact on the cell cycle. Further, that MCL1 regulation of the cell cycle is RB-1 dependent.

### MCL1 promotes growth, not G_2_/M blockage

**Fig. 7:**
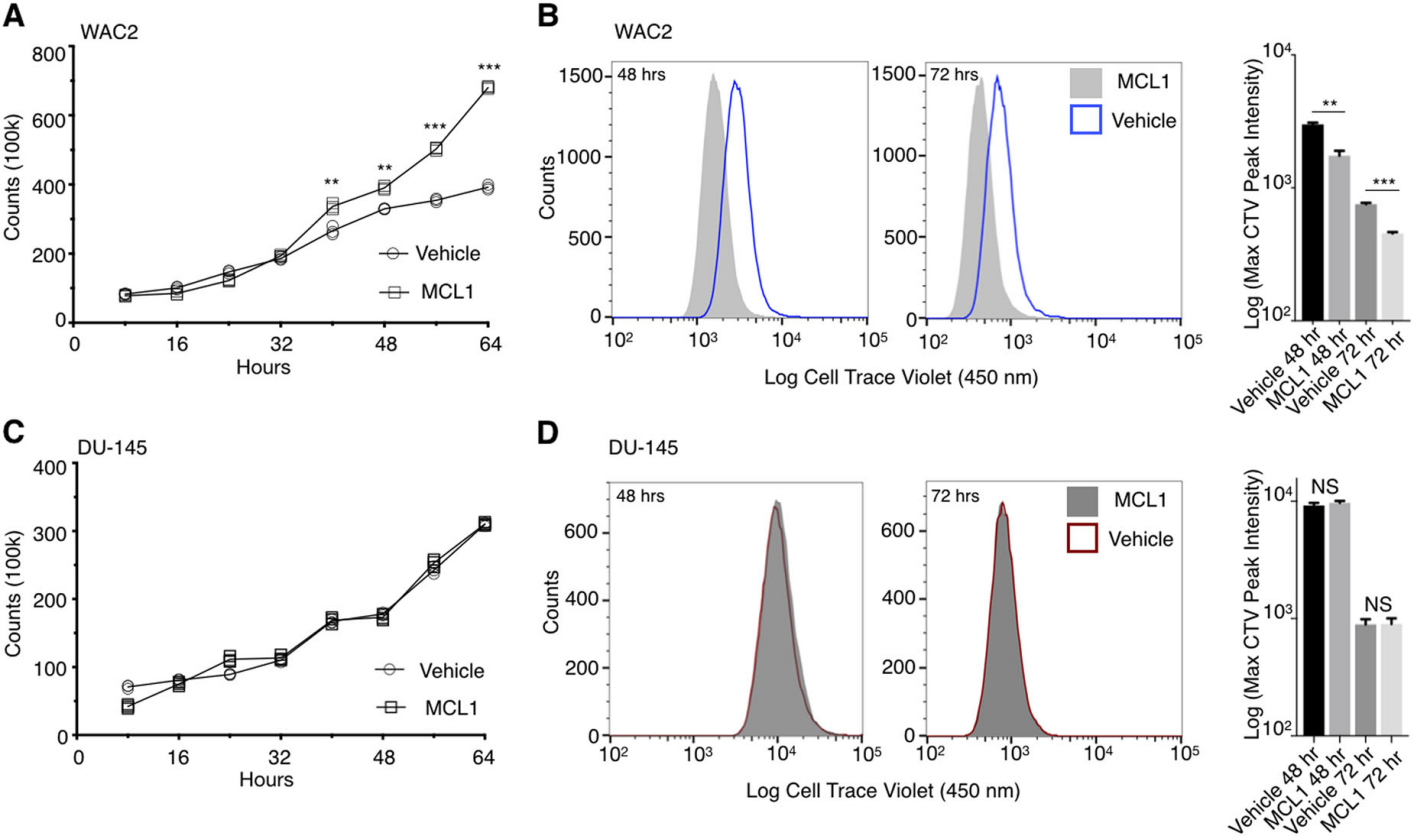
MCL1 promotes proliferation in a RB-dependent fashion. Cell proliferation was measured using cell counting in WAC2 (**a**) and DU-145 (**c**) cell lines over 64 h following vehicle or MCL1 transfection. Additionally, the dye dilution assay, Cell Trace Violet, was used to visualize proliferation in WAC2 (**b**) and DU-145 (**d**) lines at 48 and 72 h post vehicle or MCL1 transfection. Analysis of the max peak intensity for the CTV assays is shown to the right of each pair. All data are presented as mean ± S.D., *N* = 3. The statistical significance was determined by unpaired Student *t*-test where *P > 0.05; **P > 0.01; ***P > 0.001.

In the preceding studies, we focused on the effect that MCL1 has on the G_1_ cell population, yet, in cell cycle studies, a change in one group must result in redistribution to another portion of the cell cycle. We observed that overexpression of MCL1 and subsequent decrease in G_1_ resulted in a concurrent increase in the percent of cells in G_2_/M. Prior studies have observed this change in PI histograms and attributed it to MCL1 inducing a block in G_2_/M progression. Given our observation that MCL1 influences P18 protein we surmised that such an increase could be caused by either a G_2_/M block or alternatively through an increase in cell proliferation. To assess which is occurring and resulting in the change in PI histograms, we tracked cell proliferation in two ways. First, we tracked cellular growth over a 64 h time period. We observed that RB1-positive cells all exhibited increased growth rates following overexpression of MCL1, while RB1-negative cells show no change (Fig. 7a, c, respectively). Secondly, we used the cellular dye dilution marker, CellTrace™ Violet (CTV), to track proliferation^32–34^ (Supplementary Fig. S9). In this assay, as cells divide the dye is divided in daughter cells causing a decrease in dye measurement and increase in cell number. We observed that MCL1 overexpression in RB1-positive cells induced both an increase in cell number and decrease in CTV staining, consistent with increased proliferation compared to controls (Fig. 7b and Supplementary Figs. S7, S5B, S10). Conversely, RB1-negative cells exhibited no such change upon MCL1 overexpression (Fig. 7d). This strongly suggests that the observed decrease in G_1_ and increase in S or G_2_/M cell populations from MCL1 overexpression is not evidence of a cell cycle blockade but is rather due to an increase in cellular proliferation.

## Discussion

Since the discovery of the importance of BCL2, first in tumorigenesis and later for its regulation of apoptosis, considerable effort has been employed to understand the complex network of protein-protein interactions that comprise BCL2 family regulation and coordination of cell death^2,35^. Interspersed in these studies of the BCL2 family, a number of papers have highlighted how non-BCL2 family proteins can affect the anti-apoptotic BCL2 family members^36–38^. While most of these interactions have been shown to act in a pro-apoptotic fashion, some interactions have suggested a connection between the BCL2 family and other cellular homeostatic mechanisms, including the cell cycle^39–44^.

Indeed, for as long as the BCL2 family has been studied and due to this protein family’s direct involvement in regulating cellular viability, the role of the BCL2 family in cell proliferation has been surmised at being at the very least a secondary effect of maintained viability^45^. However, it has become increasingly evident that the BCL2 family may have a much more direct role in proliferation through interactions with cell cycle machinery and BCL2 family presence throughout the cell cycle^46^. Indeed, the anti-apoptotic side of the BCL2 family seemingly hands off its pro-survival role throughout the cell cycle between its members. Starting in G_1_, both BCL2 and BCLxL have been shown to maintain cellular viability in G_0_ and G_1_^40,47,48^. Beyond sustaining cell viability during interphase, these two proteins have also been shown to prolong G_1_, delaying the transition to S phase in the presence of intra-cellular stress^49^. This is borne out by the observations of a recent paper^50^ where in the specific natural killer (NK) cell type; there is a difference in protein expression of BCL2 family members in cycling versus non-cycling cells. Specifically, BCL2 was observed more in non-cycling cells and MCL1 was observed more in cycling NK cells. Moving past G_1_ into S phase, the key G_1_/S transition transcription factor, E2F1, is known to directly suppress the *MCL1* promoter^51^ and also suppresses both BCL2 RNA and protein levels^52^. Conversely, BCL2 delays the G_1_/S transition through inhibition of E2F1^40^. However, MCL1’s role is far from clear as MCL1 expression is correlated with an increase of the known G_1_/S transition protein inhibitor, p27, in neural progenitors^53^. Further, MCL1 has been shown to interact with PCNA, a DNA sliding clamp involved in processivity during S phase^39^. PCNA is a promiscuous protein with many binding partners, including p21, CDK2/4/5/6, cyclin D1, and others^54^. Fujise and colleagues observed that MCL1 is able to bind PCNA using yeast two hybrid and overexpression constructs and that MCL1 overexpression induces a decrease in the percent of cells in S phase as shown through BRDU uptake^39^. Conversely, Jamil et al.^55^ observed that following cell cycle blockade, CDK1 binds to an alternative form of murine MCL1 (snMcl-1) and that overexpression of this form slows cell growth. Despite these contradictory observations of MCL1’s role in the G_1_/S transition, its involvement cannot be ignored, and further studies are needed to disentangle MCL1 within the context of proliferative regulation at the G_1_/S checkpoint.

Our present study demonstrates the existence of a direct protein-protein interaction between the BCL2 family, through MCL1, and the G_1_/S CDK4/6-RB1 checkpoint, through P18. This interaction is mediated by a rBH3 motif found in P18. Beyond the specific interaction of MCL1 and P18, this validates the rBH3 as a native protein motif capable of mediating protein-protein interactions with the anti-apoptotic BCL2 family. Further, we show that this interaction occurs with biologically relevant affinity as it can suppress the association of the native MCL1 target, the BH3 motif of BAK.

The result of this direct protein-protein interaction was unanticipated. We initially hypothesized that P18 association with MCL1 would behave in a pro-apoptotic fashion as a parallel growth suppression signal. This would mirror recent studies that have found other stress-response proteins that are able to interact with anti-apoptotic BCL2 family proteins to directly promote cell death^56^. We observed that overexpression of P18 has no impact on cell viability. Rather, the association of MCL1 with P18 induces P18 degradation through a cysteine-protease dependent pathway.

With the observed impact on P18 protein expression, we sought to determine how P18 loss might affect cell progression through the G_1_/S checkpoint. Typically, P15 and P16 serve as the primary negative regulators of CDK4/6, yet in human cancers, overexpression of P18 has been shown to compensate for loss of P15 and/or P16^24^. Limited studies have been performed to characterize the effect of P18 loss alone, as it is not commonly observed. We therefore carried out knockout studies that demonstrate that the loss of P18 is able to promote cellular proliferation. We further observe that the loss of P18 protein induced by MCL1 overexpression can mimic this effect. This led us to determine how MCL1 is affecting the cell cycle and cell proliferation. We observed, in agreement with a number of prior studies, that increased expression of MCL1 leads to an increase in the cell population in G_2_/M. While prior studies had suggested that MCL1 upregulation induces a G_2_/M blockage based on these findings, we suspected that this was rather a result of MCL1 driving the cell through the G_1_/S checkpoint and promoting proliferation. We confirmed this hypothesis using both cell growth studies and the CellTrace™ Violet dye dilution assay. This observation synergizes with prior studies that have highlighted the role of MCL1 in regulating apoptosis during the cell cycle, especially during mitosis^51,53,57–59^. Our results suggest that having a sufficient amount of free MCL1, indicative of a non-apoptotic state, can promote cell growth and may aid in insuring that there is sufficient MCL1 to enable exit from mitosis once the cell cycle is complete. Significantly, this study is the first demonstration that MCL1 can directly initiate cell proliferation.

While we expect the protein-protein interaction between P18 and MCL1 can occur in any cell line or type where these two proteins are present, the effect on cell cycle control will likely be cell type specific. For instance, we observed a much larger impact on G_1_ population in WAC2 cells following MCL1 upregulation than observed following treatment with si-P18. This suggests that MCL1 may target multiple cell cycle regulatory proteins. Yet, treatment with a small-molecule targeting the BH3 pocket of MCL1 successfully rescued this effect completely. This suggests that other BH3 or rBH3 interactions may be present that mediate MCL1 control over the cell cycle. Conversely, we observed that in RB mutant cells the MCL1 mediated degradation of P18 occurs but does not induce cellular proliferation. In a similar manner to BH3 mimetic suppression of exogenous MCL1 induced proliferation, we would expect that upregulation of pro-death BH3-only proteins that target MCL1 to suppress this proliferative effect. This has possibly significant implications in the deployment of recently developed MCL1-targeted BH3 mimetics that are currently entering the clinic.

Finally, we expect this communication between the BCL2 family and the CDK4/6-RB pathway to exist beyond the realm of cancer and may have significant impact in normal cellular proliferation, stem cell growth, and differentiation. Specifically, investigation on how this interaction impacts hematopoietic and neuronal progenitor cell speciation, where MCL1 has previously been identified as a key mediator of differentiation, is of particular interest. In conclusion, we have established a protein-protein interaction that creates a direct communication mechanism to couple cell death and cell proliferation without the need for intervening transcription factor activation or protein translation.

## Supplementary information

Supplemental Figure Captions

Figure S1

Figure S2

Figure S3

Figure S4

Figure S5

Figure S6

Figure S7

Figure S8

Figure S9

Figure S10

Table S1
