# Supplementary material for: MCL1 binding to the reverse BH3 motif of P18INK4C couples cell survival to cell proliferation
Source: Cell Death Dis. 2020 Feb 28;11(2):156. doi: 10.1038/s41419-020-2351-1 (PMC7048787; doi:10.1038/s41419-020-2351-1)
Supplement: Supplementary file 1 — Supplemental Figure Captions [file 41419_2020_2351_MOESM1_ESM.docx]

**Supplemental Information**

**Figure S1. MCL1 and P18 Endogenous coIP.** (A) Western blot of endogenous coIP of MCL1 (IP) with MCL1 (IB), BAX (IB), and P18 (IB) in PC3. Cells harvested after 48 hour 100nM calcitriol treatment. (B) Western blot of endogenous co-immunoprecipitation of P18 (IP) with MCL1 (IB), CDK6 (IB) and P18 (IB) in PC3. Cells harvested after 48 hour 100nM calcitriol treatment.

**Figure S2.** **P16 more highly deleted than P18.** Cbioportal Oncoprint for INK4 Family, select all listed studies (zoom: 1%) Cbioportal accessed 10.23.2018

**Figure S3. P18 transfection does not induce apoptosis.** (A) Transfection of vehicle control (left) or P18 (right) (48 hr) in PC3 with Annexin V and PI staining FACs analysis. (B) Western blot confirming P18 expression transfection and overexpression. All data are presented as mean ± S.D., N=3. The statistical significance was determined by unpaired Student t-test where *P<0.05; **P<0.01; ***P<0.001.

**Figure S4. Bortezomib inversely impacts MCL1 and P18.** Western blot of bortezomib treated PC-3 cells (10, 20, or 30 uM) for 4 hours. Quantification of band intensity shown below blot. All data are presented as mean ± S.D., N=3. The statistical significance was determined by unpaired Student t-test where *P<0.05; **P<0.01; ***P<0.001.

**Figure S5.** **MCL1 modulation of cell cycle in RB+ and P16+ cell line PC3.** (A) MCL1 transfection PI FACs analysis of cell cycle population in PC3. (B) MCL1 transfection and CTV Assay in PC3. (C) cell counting assay in PC3. All data are presented as mean ± S.D., n = 3. The statistical significance was determined by unpaired Student t-test where *P<0.05; **P<0.01; ***P<0.001.

**Figure S6. Knockdown of P18.** (A) PI FACs Analysis of P18 knockdown and GFP control in WAC2, PC3 and DU-145. (B) of P18 knockdown and GFP control in WAC2, PC3 and DU-145. All data are presented as mean ± S.D., n = 3. The statistical significance was determined by unpaired Student t-test where *P<0.05; **P<0.01; ***P<0.001.

**Figure S7. P18 knockdown with cell counting proliferation assay.** (A) Cell counting assay in WAC2 undergoing P18 siRNA knockdown from 8 to 64 hours. (B) Cell counting assay in PC3 undergoing P18 siRNA knockdown from 8 to 64 hours. All data are presented as mean ± S.D., n = 3. The statistical significance was determined by unpaired Student t-test where *P<0.05; **P<0.01; ***P<0.001.

**Figure S8. MCL1 decreases P18 protein level in RB null cell line.** Western Blot of MCL1 overexpression in DU145.

**Figure S9. Cell trace violet time course.** CTV time course in untreated WAC2 cells.

**Figure S10. MCL1 modulation of cell proliferation in 3 cell lines at 0 and 24 hours.** (A) MCL1 transfection and CTV FACs analysis of cell cycle population in WAC2 at 0 hour (left) and 24 hour (right). (B) MCL1 transfection and CTV FACs analysis of cell cycle population in PC3 at 0 hour (left) and 24 hour (right). (C) MCL1 transfection and CTV FACs analysis of cell cycle population in RB mutant DU145 at 0 hour (left) and 24 hour (right).

**Supplemental Table 1. P16 and P18 ankyrin table.** P16 and P18 truncation and chimera Protein sequences organized by ankyrin repeat with bolded rBH3.
