# Supplementary figures and images for: MCL1 binding to the reverse BH3 motif of P18INK4C couples cell survival to cell proliferation
Source: Cell Death Dis. 2020 Feb 28;11(2):156. doi: 10.1038/s41419-020-2351-1 (PMC7048787; doi:10.1038/s41419-020-2351-1)

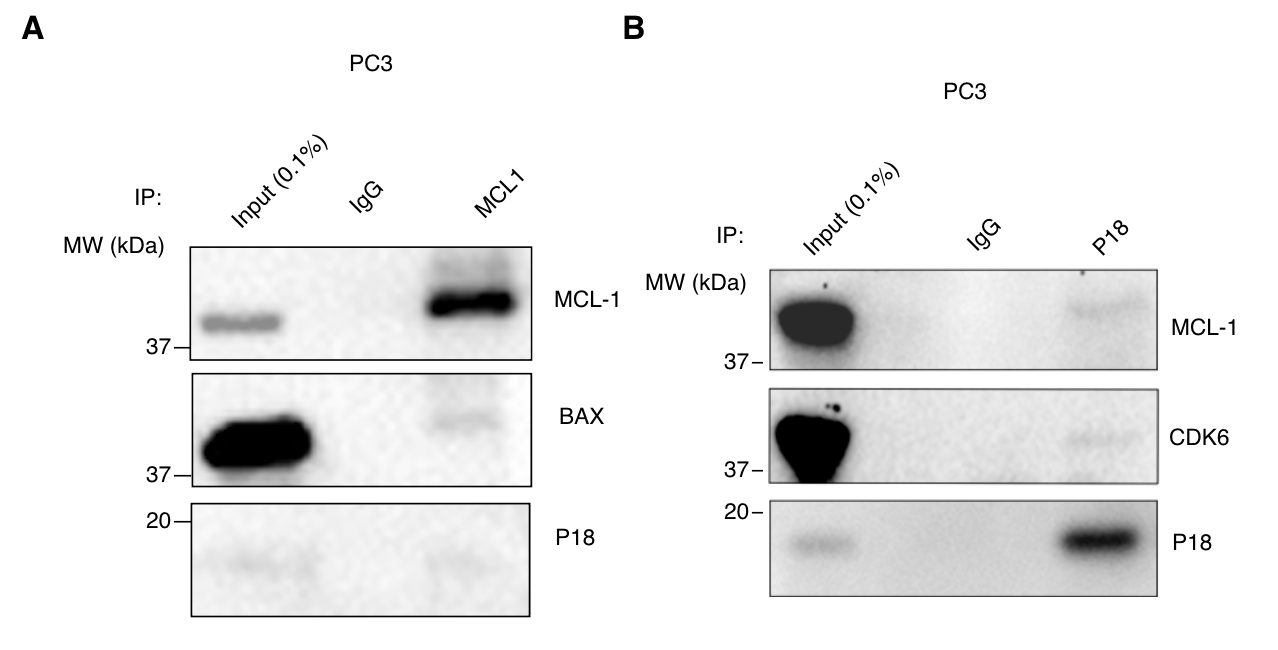

Supplement: Supplementary file 2 — Figure S1 [file 41419_2020_2351_MOESM2_ESM.png]

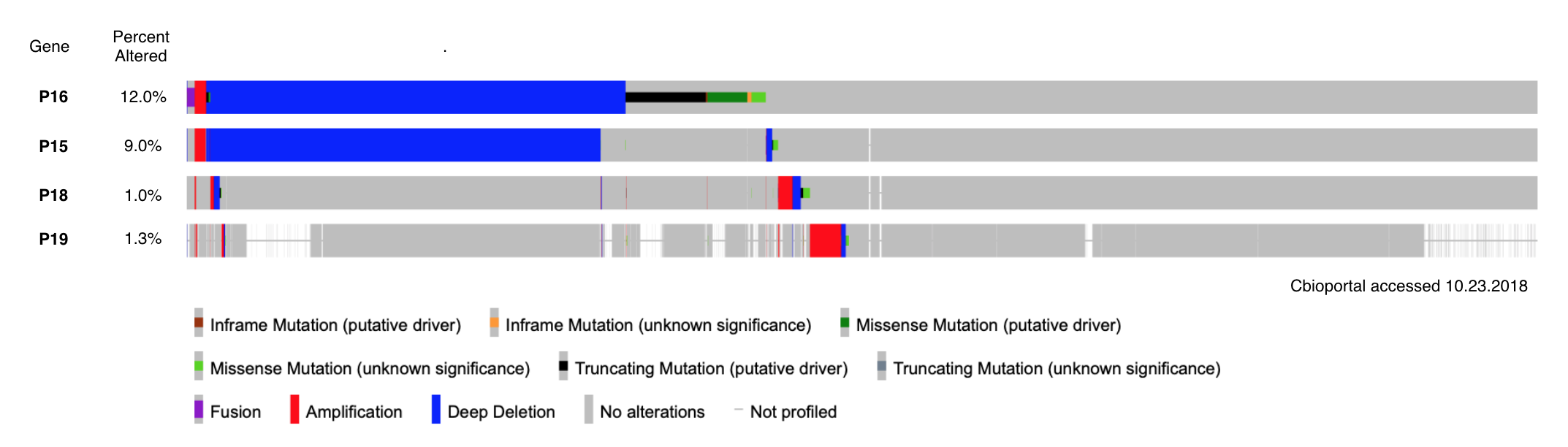

Supplement: Supplementary file 3 — Figure S2 [file 41419_2020_2351_MOESM3_ESM.png]

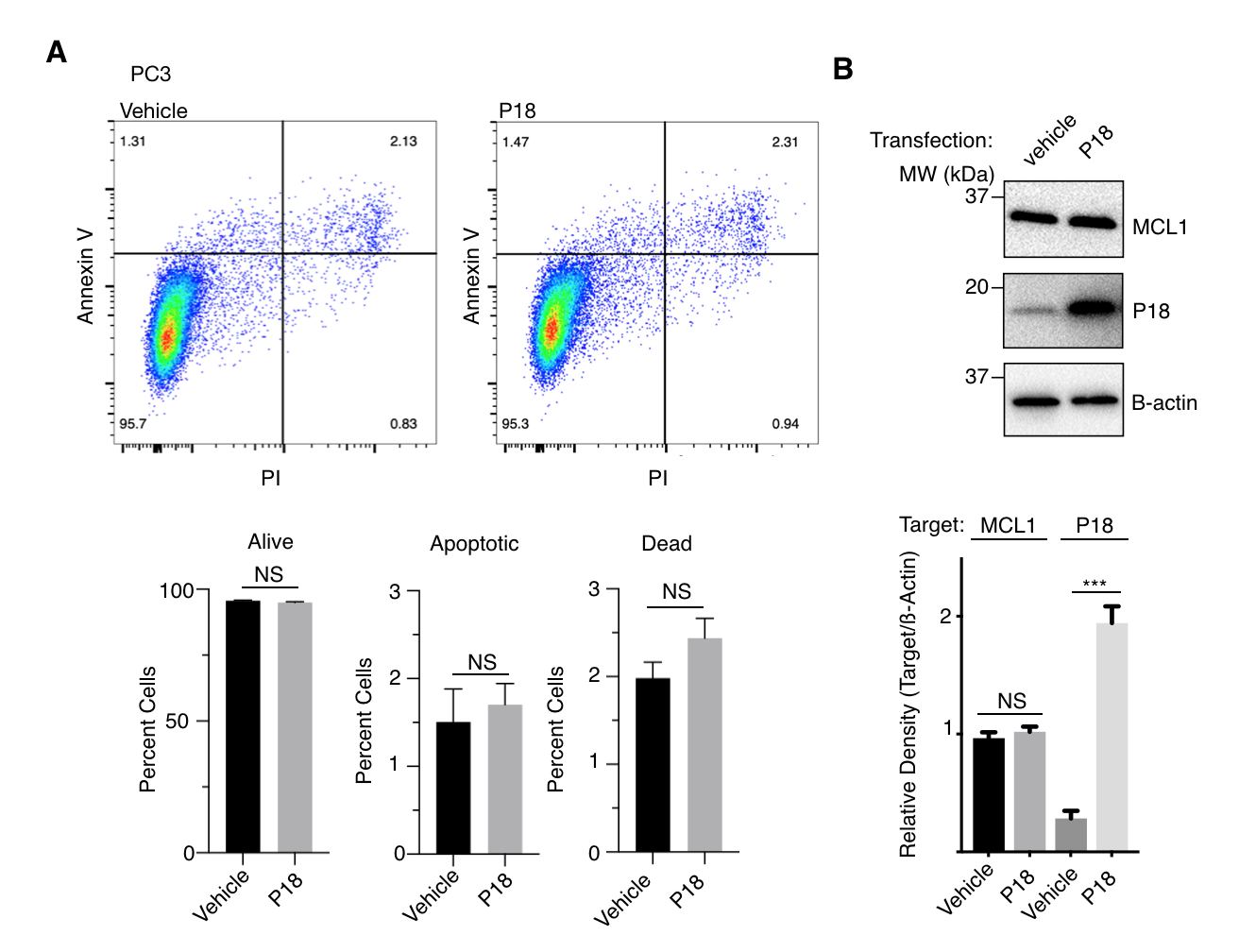

Supplement: Supplementary file 4 — Figure S3 [file 41419_2020_2351_MOESM4_ESM.png]

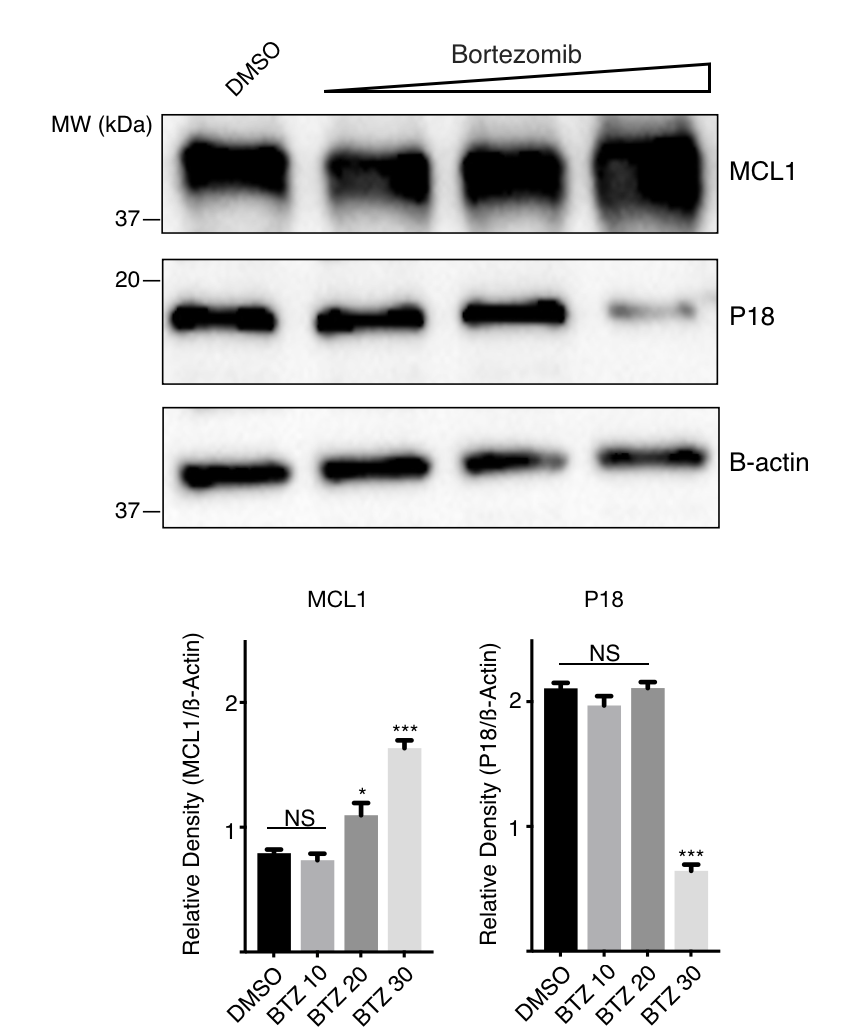

Supplement: Supplementary file 5 — Figure S4 [file 41419_2020_2351_MOESM5_ESM.png]

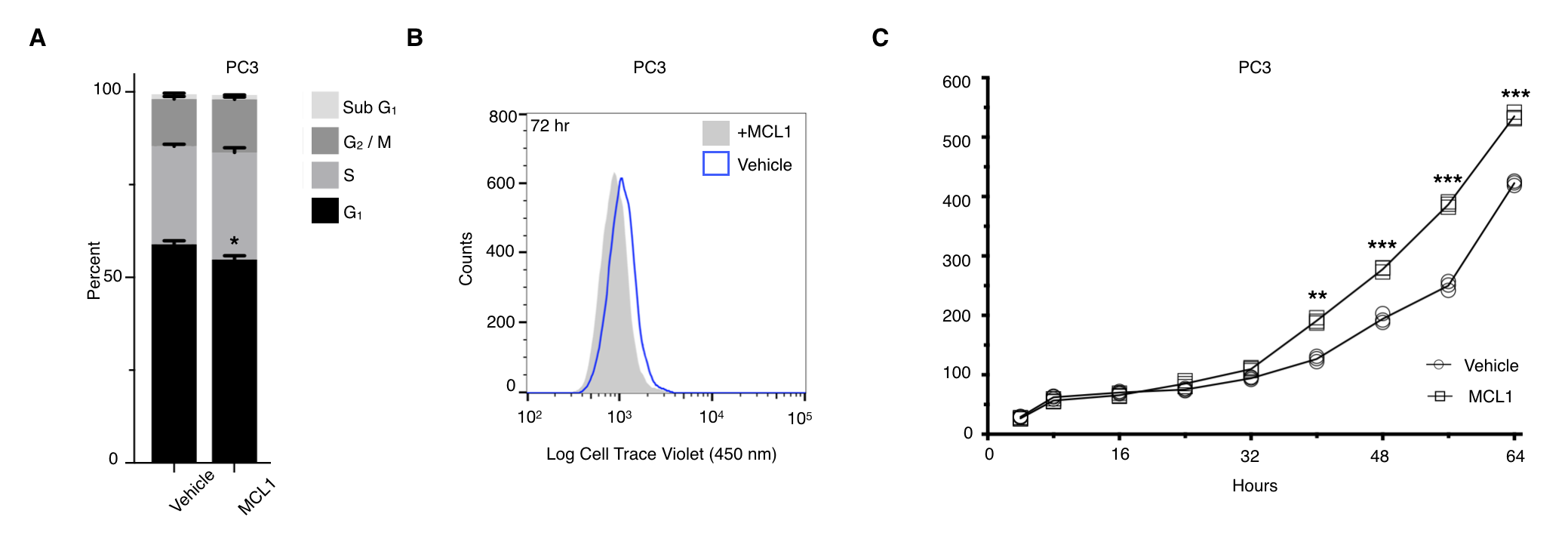

Supplement: Supplementary file 6 — Figure S5 [file 41419_2020_2351_MOESM6_ESM.png]

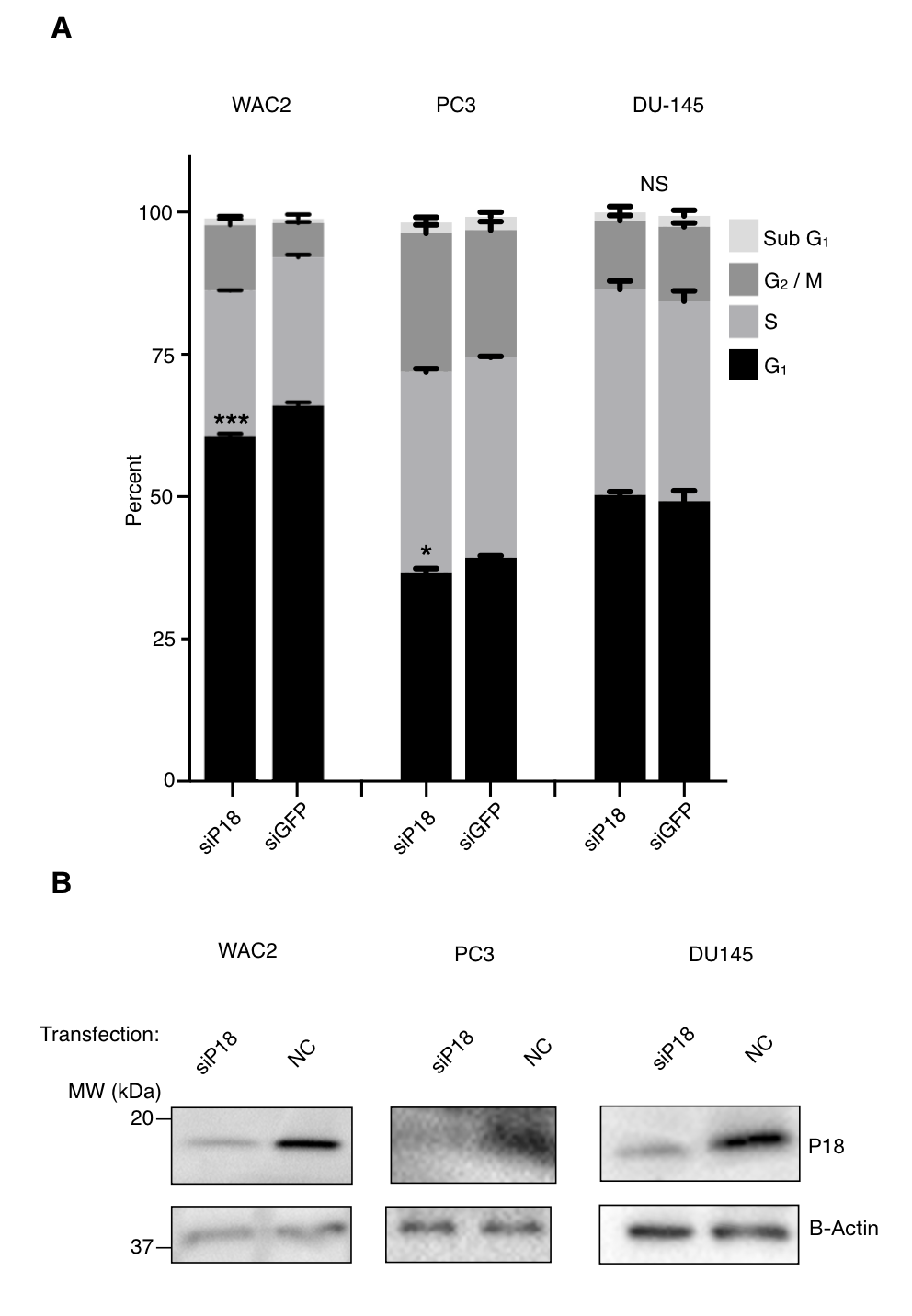

Supplement: Supplementary file 7 — Figure S6 [file 41419_2020_2351_MOESM7_ESM.png]

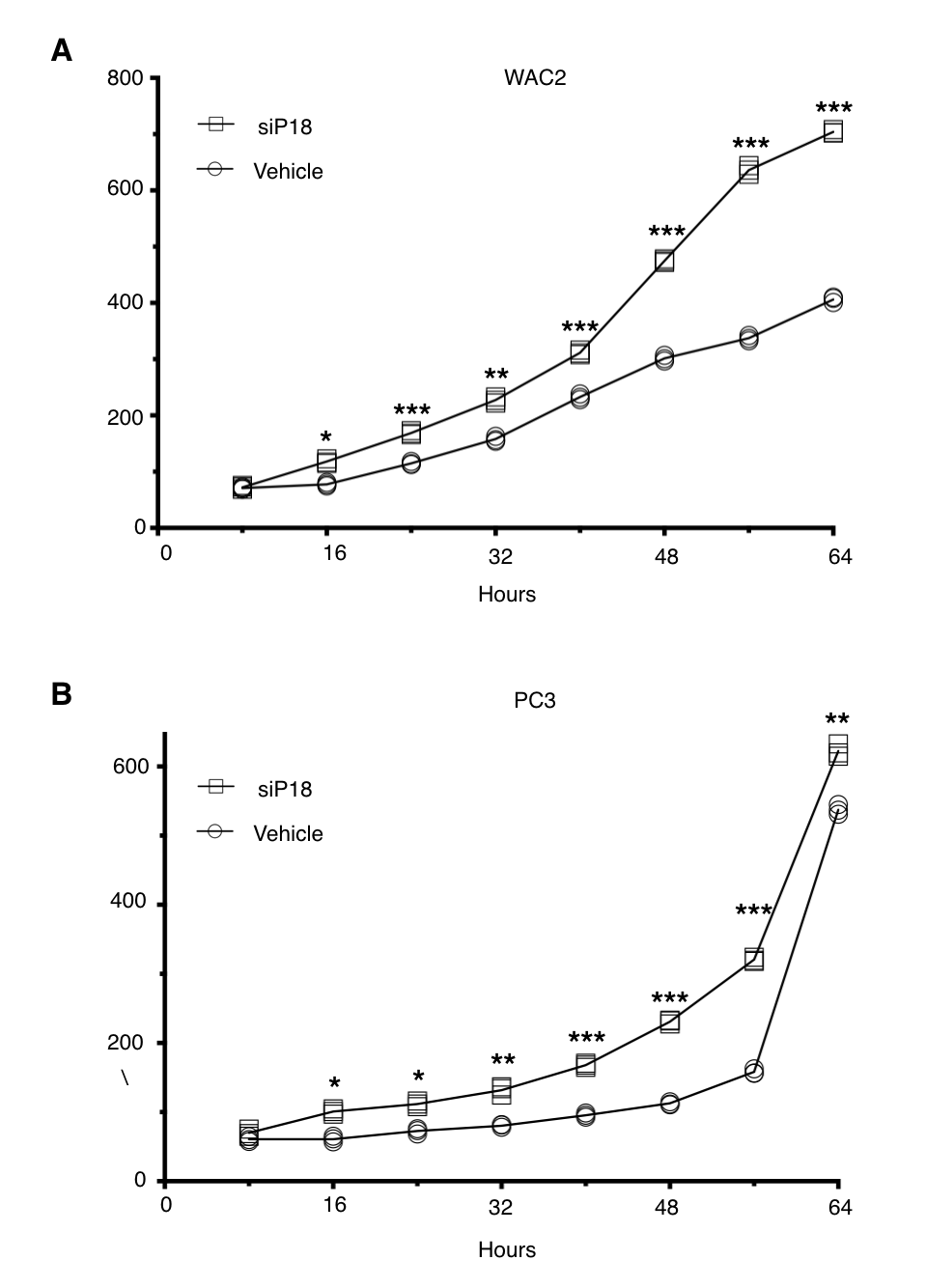

Supplement: Supplementary file 8 — Figure S7 [file 41419_2020_2351_MOESM8_ESM.png]

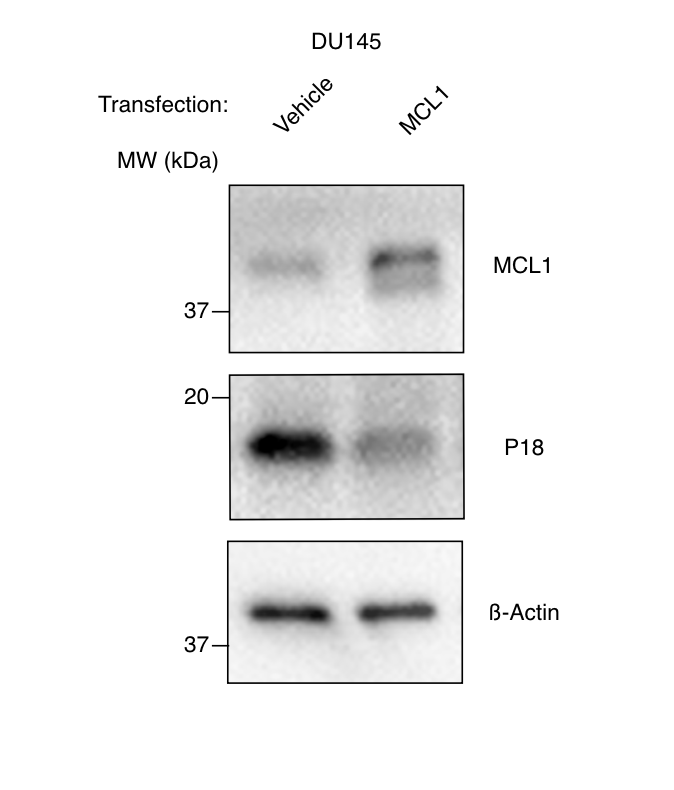

Supplement: Supplementary file 9 — Figure S8 [file 41419_2020_2351_MOESM9_ESM.png]

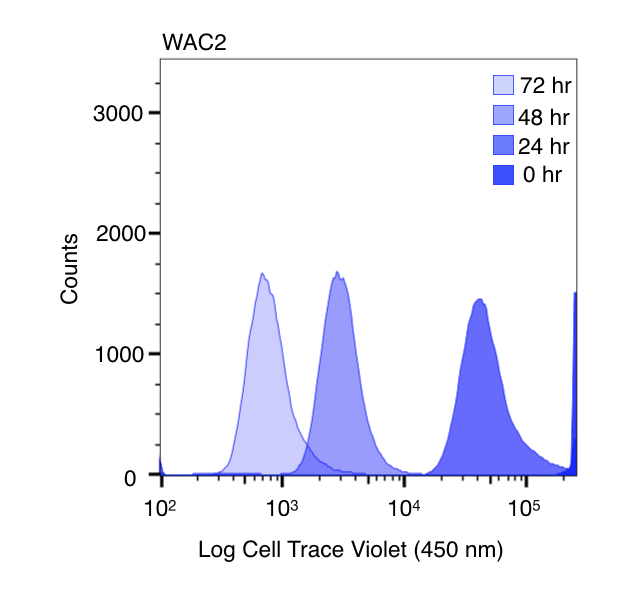

Supplement: Supplementary file 10 — Figure S9 [file 41419_2020_2351_MOESM10_ESM.png]

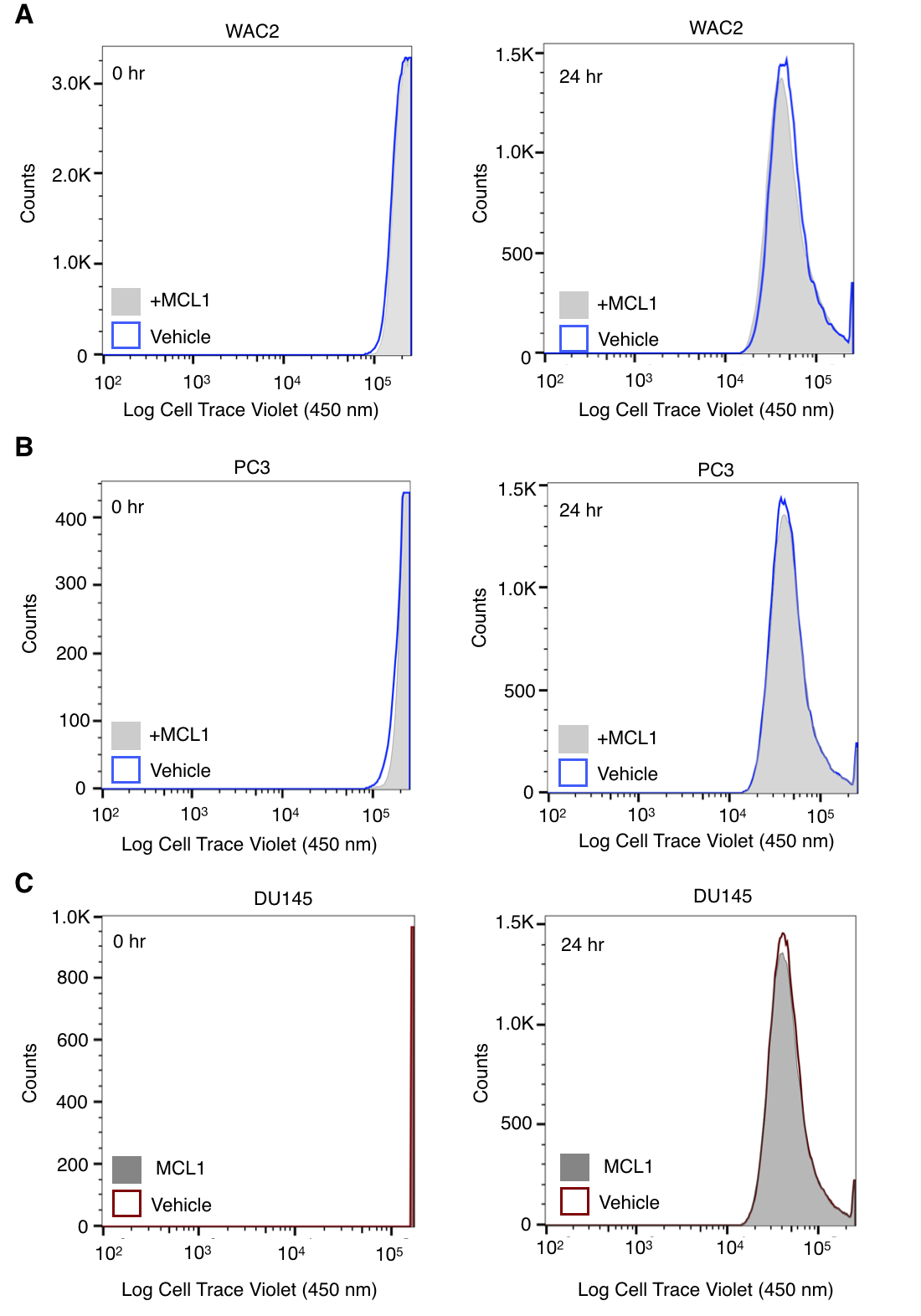

Supplement: Supplementary file 11 — Figure S10 [file 41419_2020_2351_MOESM11_ESM.png]
