# Supplementary material for: MCL1 binding to the reverse BH3 motif of P18INK4C couples cell survival to cell proliferation
Source: Cell Death Dis. 2020 Feb 28;11(2):156. doi: 10.1038/s41419-020-2351-1 (PMC7048787; doi:10.1038/s41419-020-2351-1)
Supplement: Supplementary file 12 — Table S1 [file 41419_2020_2351_MOESM12_ESM.docx]

| **Protein** | **Ankyrin 1** |  | **Ankyrin 2** |  | **Ankyrin 3** |
| --- | --- | --- | --- | --- | --- |
| **P18** | 7 NELASAAARGDLEQLTSLLQNNVNVNAQNGFGRT |  | ALQVMKLGNPEIARRLLLRGANPDLKDRTGFA |  | VIHDAARAGFLDTLQTLLEFQADVNIEDNEGNL |
| **C1-4** | 7 NELASAAARGDLEQLTSLLQNNVNVNAQNGFGRT |  | ALQVMKLGNPEIARRLLLRGANPDLKDRTGFA |  | VIHDAARAGFLDTLQTLLEFQADVNIEDNEGNL |
| **C4-5** |  |  |  |  |  |
| **P16** | 14 DWLATAAARGRVEEVRALLEAGALPNAPNSYGRR |  | PIQVMMMGSARVAELLLLHGAEPNCADPATLTR |  | PVHDAAREGFLDTLVVLHRAGARLDVRDAWGRL |
| **AC** | 14 DWLATAAARGRVEEVRALLEAGALPNAPNSYGRR |  | PIQVMMMGSARVAELLLLHGAEPNCADPATLTR |  | PVHDAAREGFLDTLVVLHRAGARLDVRDAWGRL |
|  |  |  |  |  |  |
| **Protein** | **Ankyrin 4** |  | **Ankyrin 5** |  |  |
| **P18** | 106 PLHLAAKEGHLRVVEFLVKHTASNVGHRNHKGDTA |  | CDLARLYGR**NEVVSLMQANGA**GGATNLQ |  |  |
| **C1-4** | 106 PLHLAAKEGHLRVVEFLVKHTASNVGHRNHKGDTA |  |  |  |  |
| **C4-5** | 106 PLHLAAKEGHLRVVEFLVKHTASNVGHRNHKGDTA |  | CDLARLYG**RNEVVSLMQANGA**GGATNLQ |  |  |
| **P16** | 114 PVDLAEELGHRDVARYLRAAAGGTRGSNHARIDA |  | AEGPSEMIGNHLWVCRSRHA |  |  |
| **AC** | 106 PLHLAAKEGHLRVVEFLVKHTASNVGHRNHKGDTA |  | CDLARLYGR**NEVVSLMQANGA**GGATNLQ |  |  |

**Table S1. P16 and P18 Ankyrin Table.** P16 and P18 truncation and chimera

Protein sequences organized by ankyrin repeat with bolded rBH3.
